# Injectable Scaffolds Enriched with Silver to Inhibit Bacterial Invasion in Tissue Regeneration

**DOI:** 10.3390/ma12121931

**Published:** 2019-06-15

**Authors:** Chiara Ceresa, Letizia Fracchia, Alice Marchetti, Maurizio Rinaldi, Michela Bosetti

**Affiliations:** Department of Pharmaceutical Sciences, Università del Piemonte Orientale “A. Avogadro”, Largo Donegani, 2–28100 Novara, Italy; chiara.ceresa@uniupo.it (C.C.); alicemarchetti9@gmail.com (A.M.); maurizio.rinaldi@uniupo.it (M.R.); michela.bosetti@uniupo.it (M.B.)

**Keywords:** wound healing, tissue repair, injectable biomaterials, antibacterial activity, silver, collagen, alginate

## Abstract

During wound healing, bacterial infections may prolong skin regeneration and tissue repair, causing delayed or incomplete healing. The therapeutic strategies currently used include general therapeutic modes, growth factors, skin substitutes, matrices and/or cell therapy. Among recent technologies, wound dressing materials comprising silver nitrate or silver sulfadiazine as the antimicrobial agent are widespread, despite their known cytotoxicity. The aim of this work was to develop and evaluate the efficacy of gelatinous injectable biomaterials composed of collagen and alginates, enriched with silver against bacterial pathogens commonly involved in wound infections. To reduce cytotoxicity, silver was used as lactate and saccharinated salts. Results show that silver-enriched beads were effective against both Gram-positive and Gram-negative strains in a concentration-dependent manner. Silver addition was more active against *Staphylococcus* *epidermidis* than against *Pseudomonas* *aeruginosa*. The antibacterial activity was localized only in the area of contact with the beads at concentrations lower than 0.3 mM, whereas at higher concentrations a larger inhibition halo was observed. No cytotoxic effect on eukaryotic cells was seen both testing the materials’ extracts or the Ag-doped beads in contact tests. These results, although preliminary, suggest that these scaffolds are a promising approach for realizing injectable or spreadable functional biomaterials with antibacterial activity for applications in wound management.

## 1. Introduction

Wound healing is a complex process that can be delayed by microbial infections. The presence of necrotic tissue and the lack of continuity of the damaged skin is a perfect medium for the proliferation of microorganisms [1]. Microbial infection in wounds causes a lack of healing and leads to chronicity and, in some cases, to the worsening of the wound. Even more serious is the presence of bacterial biofilms (complex communities of living microbial cells surrounded by a protective extracellular polymeric matrix) on the lesion that form colonies, causing delay in healing [2,3].

When the wound is infected, the body responds by activating an inflammatory response characterized by the release of cytokines and growth factors with vasodilation and increased blood flow to the wounded area. Phagocytes are also activated to remove microorganisms and toxins. When the lesion becomes chronic, a massive inflammatory response is initiated which, through the activation of neutrophils, causes damage to the host through the release of oxygen free radicals [4]. Several factors affect the development of bacterial infection, such as the concomitant use of drugs, the advanced age of the patient, poor circulation, diabetes, obesity, compromised or suppressed immune system, decreased mobility or immobility, malnutrition, poor hygiene and general health conditions [5]. Other factors depend on the characteristics of the wound, such as position and extension.

Complications of infected wounds can vary from local to systemic. A non-healing wound is often a cause of pain, malaise and psychological detriment for the patient. Moreover, systemic complications may occur, including bacterial infection in different sites such as the dermal or subcutaneous layers of skin, the bone or bone marrow and blood [5]. The diagnosis and management of wound infection is controversial, and varies between clinicians [1]. Most infected wounds are caused by bacterial colonization, originating either from the normal skin microflora [6], or bacteria from other parts of the body or the outside environment (i.e., airborne exogenous microorganisms or those introduced by traumatic injury) [7].

Wound colonization is most frequently polymicrobial, involving several potentially pathogenic microorganisms, either aerobic or anaerobic, that originate mostly from skin and from mucosal surfaces, such as those of the oral cavity and gut. The most common infection-causing bacteria is *Staphylococcus aureus* and other species of staphylococci, such as *Staphylococcus epidermidis*, which are part of the normal skin microflora [8,9]. Other members of the skin microbiota that may be wound contaminants are micrococci, skin diphtheroids, and propionibacteria. In addition, the gastrointestinal, oropharyngeal, and genitourinary mucosae are endogenous sources that supply the vast majority of microorganisms colonizing wounds in close proximity to those sites. Nevertheless, according to the majority of wound care practitioners, the primary causes of delayed healing and infection in both acute and chronic wounds related to the colonization of aerobic or facultative pathogens such as *Staphylococcus aureus*, *Pseudomonas aeruginosa*, and beta-hemolytic streptococci [8].

The therapeutic strategies currently used include general therapeutic approaches, addition of growth factors, use of skin substitute matrices and/or cell therapy [10]. There is a great need for a readily available, easily stored and temporary substitute for human skin for the effective treatment of different form of skin loss and thermal burns. Epidemiologically, the wound healing sector is an important part of the health care pool. An analysis carried out using Medicare’s data for the year 2014 showed that 8.2 million people in USA (15% of population) is affected by wounds (4% surgical infections and 3.4% diabetic ones). The estimated costs for the treatment range from $28.1 to $96.8 billion and the costs vary according to the type of service that performs it. In fact, while the costs for hospital treatment are around $4.9 billion, the outpatient level is around $9.9 billion, indicating how these structures are important for the continuation and advancement of proper therapy [11]. This suggests that it is essential to find new treatment options aimed at reducing health costs, but above all at improving the quality of life and compliance of patients with wound injuries. Among recent technologies, wound dressing materials reducing difficulties related to grafts and enrichment with silver nitrate or silver sulfadiazine as antimicrobial agents are widespread, despite their known cytotoxicity [12]. The aim of this work was to explore two gel type of skin dressings that we think especially suitable for application to irregular body surfaces thank to their jelly consistency. Moreover, their association with silver as antimicrobial agent has been explored. We studied two injectable gelatinous biomaterials, collagen and alginate, and trying to reduce cytotoxicity of silver as antimicrobial agent, we tested the addition of two silver salt formulations, lactate and saccharinate, that in recent studies in peri-implantitis [13] and in vaginal infections [14] have shown great efficacy on different bacterial strains with minimal toxicity on human cells. We tested *in vitro* the bactericidal activity and cytotoxicity of the two jelly formulations added to the silver salts at different concentrations. The cytotoxic effect was studied on keratinocytes and fibroblasts, as they are representative of the reparative processes and are the cellular populations most involved in wound healing. At the same time, the antimicrobial effect was studied on two bacterial strains, *Pseudomonas aeruginosa* and *Staphylococcus epidermidis*, which are involved in the infection and often cause a delay in the tissue repair process. This approach was designed to optimize the antimicrobial activity of the 3D jelly scaffold suitable for application into a skin wound dressing to promote the healing process.

## 2. Materials and Methods

### 2.1. Collagen and Alginate Beads Preparation

Stocks of silver lactate or silver saccharinate (300 mM) were prepared starting from salts (Sigma-Aldrich (Milan, Italy)) diluted in culture medium specifically for eukaryote cell lines used for cytotoxicity tests and stored at 4 °C. Before the experiment, stock solutions were sonicated to obtain homogeneous suspension and serial dilution was done in collagen or alginate.

For collagen, a collagen gel (3.5 mg/mL) was obtained according to Elsdale et al. [15] from rat tail tendons (approved by the Ethics Committee for Animal Testing of the Università del Piemonte Orientale “Prot. 10825-10.07.2013”). Tails were recovered at the end of the trial and processed for collagen extraction. Briefly, the skin was skinned, the superficial vessels were removed and, starting from the tip towards the base of the tail, the vertebrae were disarticulated by removing the inserted tendons. The isolated tendons, reduced in size to about 0.5 mm^2^, were left to dry in air for 3–4 h. Afterwards, the material was weighed and sterilized by exposing it for 48 h to UV rays and then transferred to a sterile solution of acetic acid at 1‰ with a ratio of 300 mL of acetic acid per 1 g of tendon. After 48 h of stirring at 4 °C, a solution was obtained, composed of dissolved collagen and undissolved residual fibers, removed by filtration on sterile gauze. The filtrate was then centrifuged for 1 h at 16,000× *g* at 4 °C in sterile tubes and the supernatant collected and stored at 4 °C if used within a month, or at −20 °C for longer periods. Collagen produced by the procedure described above was not used in liquid form, but in the form of clusters. In a non-stick plate, a suitable quantity of 40 μL of collagen drops were created, in which a same volume of culture media containing silver at different concentrations was added. The plates were then incubated at 37 °C for 30 min, to allow collagen polymerization activation. Afterwards, the drops created were covered with abundant basal culture media for each cell line and left for another 30 min in an incubator.

For alginate, a commercial alginic acid powder (Sigma-Aldrich (Milan, Italy)) at a concentration of 1.2% was dissolved in a water solution containing 9.06 mg/mL NaCl (Sigma-Aldrich (Milan, Italy)) and sterilized by filtration. As for collagen, alginate was not used in liquid form but as clusters. To obtain a gel, the dialysis/diffusion method was used: an aqueous alginate solution was made to gel by diffusion of divalent cations from an external solution placed in contact with the former. By means of an insulin syringe, 7 μL of alginate embedded with silver lactate or silver saccharinate from 15 mM to 93 mM were dropped in a sterile solution of CaCl_2_ 15 mg/mL. The microspheres were left to crosslink in the CaCl_2_ solution for 7 min and subsequently rinsed with 0.9% NaCl solution.

Appropriate controls for each biomaterial were also prepared: positive control clusters were enriched with PenStrep 20× (antibacterial test); negative controls consisted of collagen and alginate scaffolds only.

**Figure 1:**
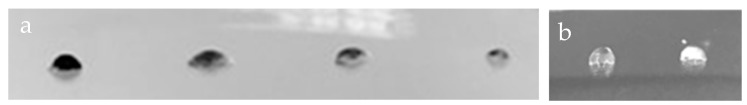
Images of collagen and alginate clusters. Collagen beads were created by two-step polymerization phases at 37 °C for 30 min (**a**). Alginate beads were formed by dropping jelly biomaterial in CaCl_2_ solution (**b**).

The silver-collagen and silver-alginate clusters and controls were taken via sterile tweezers and then used for the bacterial inhibition test or for eukaryotic viability test described below. Beads of alginate have a mean size of 17.36 ± 2.84 mm^2^ and beads of collagen have a mean size of 27.01 ± 3.88 mm^2^ (Figure 1).

A summary of the silver concentrations tested, volumes and the amount of silver contained in the two jelly biomaterials is reported in Table 1.

### 2.2. Antibacterial Activity Evaluation

Agar diffusion method was selected to verify the antibacterial effectiveness of silver-enriched collagen and alginate beads. Antibacterial activity was tested against the Gram-positive *Staphylococcus epidermidis* (ATCC 12228) and the Gram-negative *Pseudomonas aeruginosa* (ATCC 10145), based on The European Committee on Antimicrobial Susceptibility Testing (EUCAST) guidelines [16], with some modifications. Briefly, Mueller-Hinton agar was used as the bacterial growth medium. The agar surface was inoculated by using a swab dipped in a cell suspension of a 0.5 McFarland turbidity standard, approximately corresponding to 1 × 10^8^ to 2 × 10^8^ colony forming unit per ml (CFU/mL). Silver-enriched beads were placed on each *S. epidermidis* and *P. aeruginosa* inoculated agar plate by means of sterile tweezers and incubated at 37 °C for 20 h. The positive control consisted of collagen and alginate beads enriched with PenStrep 20× (Sigma-Aldrich (Milan, Italy)), negative controls were made of collagen and alginate scaffolds only. Afterwards, the inhibition zone areas were measured by QWin software (Leica) after digital acquisitions. The experiments were carried out in triplicate in four experimental replicates for both bacterial strains with regard to alginate (n = 12) and in triplicate in two experimental replicates with regard to collagen (n = 6).

### 2.3. Eukaryotic Biocompatibility

The in vitro biocompatibility of silver salts embedded in collagen and alginate clusters was evaluated using a spontaneously immortalized human skin keratinocyte line (HaCaT) and a human fibroblast cell line (MRC-5) purchased from ATCC and cultured as described. Briefly, fibroblasts were seeded and grown in Eagle’s minimum essential medium, (EMEM, EuroClone (Pero, Italy)) with 10% fetal bovine serum (FBS, EuroClone (Pero, Italy)), non-essentials aminoacids (NEAA 100×, Lonza (Basel, Switzerland)), glutamine used at 1% (200 nm, EuroClone (Pero, Italy)) and penicillin/streptomycin (PS) antibiotic used at 1% (penicillin 100 units/mL; streptomycin 10 mg/mL, EuroClone (Pero, Italy)). HaCaT (CLS 300493) were seeded and grown in Dulbecocco’s modified Eagle’s medium high glucose (DMEM HG, EuroClone (Pero, Italy)) supplemented with 10% FBS (EuroClone (Pero, Italy)), 1% glutamine (200 nm EuroClone (Pero, Italy)) and 1% PS antibiotic (penicillin 100 unit**/**mL; streptomycin 10 mg/mL, EuroClone (Pero, Italy)). Where not specified, reagents were from Sigma-Aldrich (Milan, Italy). The cytotoxicity of each silver-loaded alginate or collagen formulation was evaluated using a 3-(4,5 dimethylthiazol-2-yl)-2,5-diphenyltetrazolium bromide (MTT) (Sigma-Aldrich) assay, testing both the materials’ extracts (release tests) and the Ag-doped beads (contact tests). MRC5 and HaCaT were plated at a cell density of 1 × 10^5^ cells/well in 24-well plates, in the presence of FBS, lowered to a concentration of 4% in order to not excessively affect cell viability. After 24 h incubation at 37 °C in 5% CO_2_ atmosphere, for the contact test, one microsphere of each concentration of silver-loaded alginate or collagen formulation was added to each well. For the release test, cell-free wells were filled with 500 μL of specific medium for each cell line added to 4% FBS. Clusters of collagen or alginate enriched with silver were added at various concentrations and left for 6 h. After the time required for the clusters to release silver salts, the release medium was used to replace the one in the wells of the plate containing the previously plated cells. After 24 h of culturing in media containing 4% FBS at 37 °C, in the presence of 5% CO_2_ atmosphere, 50 µL of the MTT solution (5 mg/mL) were added to the cell monolayers, after removing microspheres in the contact tests. Plates were then incubated for 4 h at 37 °C in the dark. Formazan crystals formed during this process were extracted by adding 200 µL of HCl solution in isopropanol (250 mL of 0.05 M hydrochloric acid in 5 mL of isopropanol). After 20 min, the absorbance was measured at 570 nm using a microplate reader (Viktor X4, PerkinElmer (Waltham, MA, USA)). The experiments were carried out in duplicate in three experimental replicates for both cell lines and for both scaffolds (n = 6).

### 2.4. Statistical Analysis

The statistical analysis of the data was performed using the IBM SPSS Statistics for Windows (IBM Corp. Released 2016. Version 24.0. Armonk, NY: IBM Corp.). A multiple comparison of the data was made using analysis of variance (ANOVA), and the Bonferroni post hoc test was applied to evaluate the trend differences of the measured parameters. The *p* value was obtained from the ANOVA table and the level of 0.05 was considered to reflect the statistical significance.

## 3. Results

### 3.1. Antibacterial Activity of Injectable Scaffolds Enriched with Silver Lactate and Silver Saccharinate

**Figure 2:**
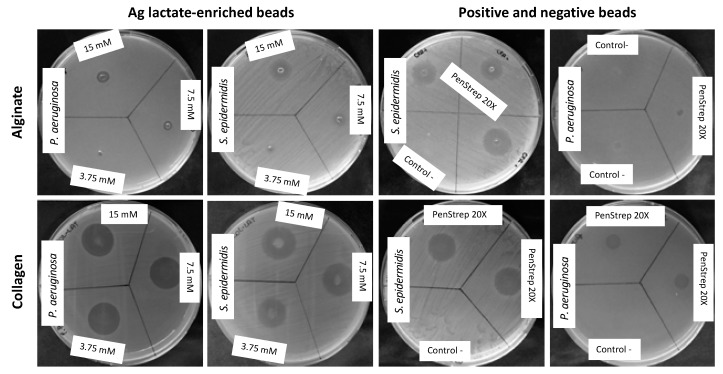
Representative images of biomaterials antibacterial activity. Alginate/collagen beads embedded with silver were placed on inoculated agar plates and incubated at 37 °C for 20 h. As positive control, beads were enriched with PenStrep 20×. Negative controls were also included to verify the inefficacy of collagen and alginate scaffolds only.

The antibacterial activity of silver lactate and silver saccharinate alginate and collagen beads was evaluated by means of the agar diffusion method (Figure 2).

**Figure 3:**
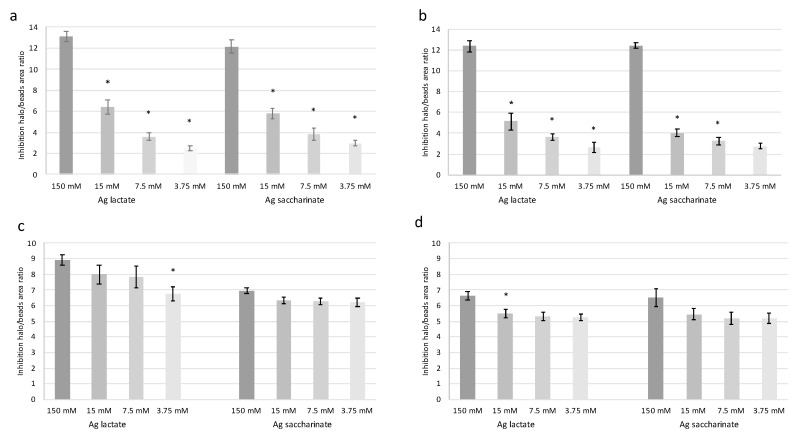
Antibacterial activity of silver evaluated by means of the agar diffusion method. Activity of silver lactate and silver saccharinate in alginate beads against (**a**) *S. epidermidis* and (**b**) *P. aeruginosa*. Activity of silver lactate and silver saccharinate in collagen beads against (**c**) *S. epidermidis* and (**d**) *P. aeruginosa*. In (**a**,**b**) * *p* < 0.05 compared to the corresponding higher concentration of silver salt (Bonferroni post hoc test). In (**c**,**d**) * *p* < 0.05 compared to the 150 mM concentrations of silver salt (Bonferroni post hoc test). Bars in the graphs indicate standard errors.

Since it was not possible to obtain clusters with a constant area, the halo of bacterial inhibition was measured in comparison to the size of the clusters and the results expressed as halo/beads ratio (Figure 3). In general, silver-enriched beads were effective against both *S. epidermidis* and *P. aeruginosa*. Moreover, according to ANOVA, all the concentrations tested were significantly different to collagen and alginate beads w/o silver (*p* < 0.01).

For alginate beads (Figure 3a,b) significantly higher activity in both silver formulations was observed at 150 mM compared to the other concentrations (*p* < 0.01). At the lower concentrations tested (15 mM, 7.5 mM and 3.75 mM), for both silver formulations and for both bacterial strains, antibacterial activity decreased in a dose dependent manner. Moreover, for each strain, no significant differences were observed between silver lactate and silver saccharinate. Both silver lactate and saccharinate at 15 mM showed higher efficacy on *S. epidermidis* than on *P. aeruginosa*, even if not significantly.

Concerning collagen beads (Figure 3c,d), in both silver formulations and for both strains, slightly higher activity was observed at the concentration of 150 mM compared to the other concentrations, although these differences were significant only for collagen beads enriched with silver lactate against *P. aeruginosa* (*p* < 0.05); on the other hand, the antimicrobial efficacy at 15 mM, 7.5 mM and 3.5 mM was generally constant and did not show significant variations, with the exception of silver lactate at 3.75 mM for *S. epidermidis* which is significantly different compared to the concentration of 150 mM. The silver lactate formulation showed a significantly higher activity on *S. epidermidis* than the saccharinate form at 150 mM (*p* < 0.01). Moreover, the silver lactate beads were also more efficient on *S. epidermidis* than on *P. aeruginosa* at all the tested concentrations (*p* < 0.001). For *P. aeruginosa,* a higher antibacterial activity of silver lactate compared to saccharinate was not detected.

Comparing the activity of both silver formulations in collagen and alginate, higher antibacterial activity was observed when silver was conveyed in collagen rather than in alginate. Although the latter showed excellent results at 150 mM, collagen was more active at all the other tested concentrations.

For antibacterial tests, the IC_50_ was calculated following a hyperbolic model through the origin (Table 2). The lowest IC_50_ values were observed when silver salts were conveyed in collagen beads with values around 1 mM, except for silver saccharinate vs. *S. epidermidis* whose IC_50_ was 0.42 mM. These results confirm that collagen clusters seem to be more active against bacteria than alginate ones, whose IC_50_ values ranged from 17.86 mM for silver saccharinate vs. *S. epidermidis* to 31.16 mM for silver saccharinate vs. *P. aeruginosa.*

### 3.2. Biocompatibility of Silver-Enriched Scaffolds on Eukaryotic Cells

In this in vitro study, we developed two 3D delivery vehicles based one on alginate and one on collagen tuned for the local delivery of silver used in form of silver lactate (SL) or silver saccharinate (SS). According to the obtained antimicrobial activity of the silver-loaded alginate and collagen, a concentration range between 15 and 3.75 mM was selected as the optimal concentration for the investigation of SL and SS cytotoxicity. A silver concentration 10-fold higher (150 mM) was used for comparison. Alginate or collagen beads alone were used as a negative control.

**Figure 4:**
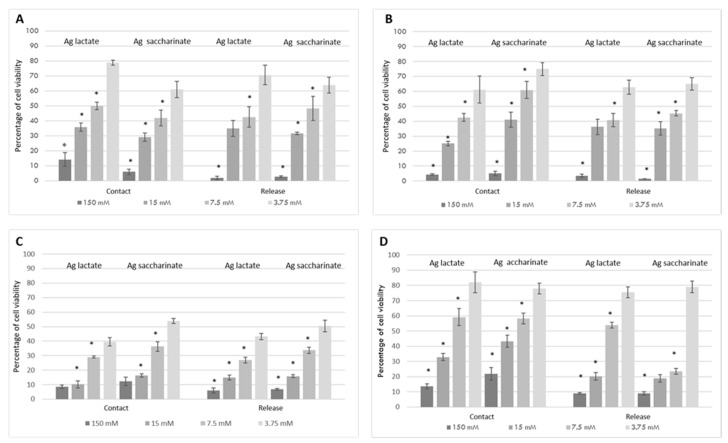
Eukaryotic biocompatibility of silver salts embedded in collagen and alginate beads evaluated by the 3-(4,5 dimethylthiazol-2-yl)-2,5-diphenyltetrazolium bromide (MTT) assay in contact tests and release tests. Cytotoxicity evaluation of silver lactate and silver saccharinate in alginate beads (**a**,**b**) and in collagen beads (**c**,**d**); results reported in (**a**,**c**) are relative to the human fibroblast cell line (MRC-5) whereas in (**b**,**d**) are reported results obtained with HaCat cells. * *p* < 0.05 compared to the corresponding lower concentration of silver salt (Bonferroni post hoc test). Bars in the graphs indicate standard errors.

After 24 h cell monolayers in contact with the silver-loaded microspheres were evaluated using MTT assay. Both HaCat and MRC5 exhibited dose-dependent cytotoxicity when in contact with SL and SS at concentrations of 150–7.5 mM (Figure 4). Regardless of the cell type assessed, cell viability started to decrease when silver was used at concentrations of 7.5 mM. After 24 h, silver-loaded alginate and collagen at 7.5 mM significantly reduced cell viability in comparison to lower concentrations, irrespective of the cell type (*p* < 0.05) or the scaffold used (Figure 4).

Studies on alginate show high cytotoxicity of silver lactate and saccharinate ions at concentrations equal to 150 mM, both testing the materials’ extracts or the silver-doped beads in contact tests with a less toxicity of Ag-lactate than Ag-saccharinate. As in the diffusion test on agar, in which the bactericidal activity appeared to be dose-dependent, the vitality test also showed a similar trend: when the concentration of silver decreased both formulations showed decreasing toxicity on MRC5 and HaCat. Of the two silver formulations, the lactate form showed a cellular vitality of MRC5 greater than the saccharinate form, and comparing the two cell lines a higher sensitivity of MRC5 for saccharinate was observed compared to keratinocytes, on which the cluster of silver lactate at the same concentration was more toxic.

Unlike alginate, in collagen there were no great differences in terms of cytotoxicity between the two formulations of silver within the same cell population (Figure 4c,d). MRC5 were the most sensitive cells that best denoted changes in cell viability. The collagen formulations proved to be more cytotoxic when compared with the same concentrations of silver-doped alginate formulations. Since collagen containing 3.75 mM of silver showed high antimicrobial activity on bacteria, we can hypothesize a continuation of the study using lower concentrations. The ultimate goal will then be to identify the lowest silver concentration to ensure microbial inhibition by maintaining cell viability values of at least 70/80% on fibroblasts. We have already begun to test even lower concentrations of silver in the scaffolds (1.87 mM), noting a good antibacterial response (not reported).

## 4. Discussion

Wound healing is a dynamic, interactive and multicellular process that often requires treatment with antimicrobial agents to avoid infections that negatively affect the tissue regeneration process. To promote the healing of skin lesions, there are more than 3000 types of commercial products, including plasters, creams and sprays. Moreover, for a correct healing process, antibacterial activity is required and, as suggested by recent studies [17], it can be obtained by enrichment with silver, avoiding undesirable effects that could derive from the use of antibiotics. Despite the extensive use of formulations containing silver in ionic, nano-crystalline or inorganic form [18,19], many doubts remain about its toxicity and about the lack of a rational relative cost-benefit analysis for the products. The ionic form is necessary to obtain cellular internalization with cytotoxicity, which is a desired effect on the prokaryotes, but not on eukaryotes. For this reason, formulations enriched with silver salts (mainly AgCl and AgNO_3_) are widely used at low dosages in creams or conveyed by low-release scaffolds in patches [20]. Ideal scaffolds for wound healing treatment should facilitate tissue healing and regeneration and possess antibacterial properties, to avoid bacterial colonization. Furthermore, an appropriate dressing, besides being biocompatible, should help reduce pain, prevent excessive fluid loss and maintain a moist environment for healing [21].

In our study, we have chosen to convey silver into a gelatinous scaffold, as it is easily moldable, deformable, able to easily integrate on the injured skin surface and to penetrate into cavities in difficult locations, with the possibility of injecting it directly into the wound. Recent studies have shown the greater efficacy of two silver salt formulations, lactate and saccharinate, on different bacterial strains, with minimal toxicity on human cells [22,23]. The in vitro bactericidal activity and cytotoxicity of these two silver salt formulations, incorporated in two injectable gelatinous biomaterials, collagen and alginate, were evaluated.

One of the two scaffolds chosen, collagen, is already known from our previous studies and from literature to be biocompatible and to actively promote integration with the cells of the host tissue [24]. Being a main component of the extracellular matrix of the dermis, it is favorably repopulated by the resident fibroblasts. The second scaffold chosen for our experiments, alginate, is not a material previously used in our laboratories, but has attracted our attention for the ease of formulation and for the presence of bibliographic data, which show excellent cellular interaction, as well as ease of use as a scaffold for the release of biomolecules and or cells [25]. Alginate is a natural hetero-polysaccharide with a wide variety of biomedical applications, as it is biocompatible, injectable, has a wide gelation capacity and low toxicity. Numerous studies have demonstrated the relevance of this biomaterial in the tissue healing process, thanks to its structural similarity with the extracellular matrix [26].

Using these gelatinous compounds as a starting point, the goal was to test new silver-based salts as antibacterial agents with less cytotoxicity on cells at the application site. To this end, we chose two saline formulations based on lactate and saccharinate, as recent in vitro studies in the vaginal and peri-implant environment [13,27] suggest their lower cytotoxicity and greater antibacterial activity compared to other commercial products containing silver.

The antimicrobial effect was studied on two bacterial strains, *Pseudomonas aeruginosa* and *Staphylococcus epidermidis*, which are commonly involved in wound infection and often cause a delay in the tissue repair process. The antibacterial activity of the two silver formulations on both strains was dose-dependent, suggesting a good ability of alginate to release silver ions, or high antibacterial activity of silver ions retained in the cluster on the inhibition of bacterial growth. Future work will be aimed at evaluating the silver dosage released by the clusters by neutron activation analysis (NAA), although we are more inclined to believe in activity at low dosages, as a low release of silver ions was detected from this kind of scaffold [13]. Silver, in fact, is historically known for its oligodynamic activity, which can be defined as the ability of small amounts of heavy metals to exert a lethal effect on bacterial cells, and among metals (gold, platinum, Cu, Zn, Ti etc.), silver is known to exert the most powerful antibacterial action [28]. In this work, all the concentrations of silver conveyed in the alginate beads appeared to be suitable for obtaining an effective bacterial inhibition on both strains.

Some studies revealed that alginate itself has its own antibacterial activity [26]. This has not emerged in our tests, as the negative controls did not provide any halo of bacterial inhibition. This could be due to the low sensitivity of the agar diffusion test used for microbial inhibition experiments.

Furthermore, the incorporation of silver into alginate dressings was found to increase antimicrobial activity and improve the binding affinity for elastase, matrix-2 metalloproteases (MMP-2) and pro-inflammatory cytokines, and to increase antioxidant activity [29].

Collagen was chosen as the second injectable biomaterial to test the antibacterial activity of silver lactate and silver saccharinate formulations. Several studies, in fact, show that collagen is widely used in wound dressing and that it is a good scaffold for the delivery of substances, thanks to its effective characteristics [30,31,32]. Collagen is a fibrous protein of muco-polysaccharide nature, which constitutes the essential component of the intercellular substance of the connective tissue. Collagen, like alginate, has multiple properties: it is a surfactant, it is biodegradable, non-toxic and more biocompatible than other natural polymers, and is only weakly antigenic. It is able to form fibers with high tensile strength and stability through cross-linking and self-aggregation. It can therefore be used as a scaffold for the transport of biomolecules.

In the current work, we observed that, unlike alginate, the antibacterial activity of silver saccharinate formulations in collagen was not dose-dependent. This could be due to the greater retention of silver ions within the collagen structure compared to alginate. Comparing the activity of silver in the two scaffolds, the best antibacterial activity was noted in collagen compared to alginate; although the latter showed excellent results at 150 mM, collagen was more active than alginate at all the other concentrations tested, as also confirmed by the IC_50_.

Collagen dressings are already found on the market for the treatment of wound healing, thanks to the previously-described properties. A study conducted on silver-enriched collagen in a nano-crystalline form has not only demonstrated the effectiveness of silver as an antimicrobial agent, but has shown that, when carried in collagen, it is able to promote the migration of fibroblasts, and therefore to promote the correct skin healing process [33]. By comparing bacterial inhibition studies with cytotoxicity studies, it can be ascertained which is the best antibacterial agent concentration that could be used in wound healing for infection prevention; it is assumed that more diluted concentrations of silver may have less cytotoxic effects on human cells, while maintaining good antibacterial activity.

Concerning the two bacterial strains tested in this work, silver-loaded alginate and collagen were efficient against both Gram-positive *S. epidermidis* and Gram-negative *P. aeruginosa*, although, in general, higher activity was observed against *S. epidermidis*. In a recent work, gelatin cross-linked nanofibers prepared via a “green electrospinning technique” doped with silver nanoparticles achieved a significant antibacterial effect against both Gram-positive and Gram-negative bacteria, where *S. epidermidis* was found to be more susceptible than *P. aeruginosa* [21].

The use of silver has been investigated as a potential risk to human health and environmental biota depending on the grade of silver used. Following their entry into systemic circulation, silver particles can migrate and induce toxicity to many organs [34]. On a cellular level, silver is internalized by macrophages and sorted to the cytoplasm. Intracellularly, released silver ions interfere with mitochondrial functions and induce apoptotic cell death. Nevertheless, eukaryotic cells show higher structural and functional redundancy than prokaryotic cells. Thus, much higher silver ion concentrations (more than 1.6 ppm Ag ions) are required to achieve comparable toxic effects in eukaryotic cells than in prokaryotic ones [35].

In our study, the cytotoxic effects of a range of silver lactate and silver saccharinate concentrations incorporated in alginate or collagen microspheres were evaluated and their effects on the viability of keratinocytes and fibroblasts were assessed, as they are representative of the reparative processes and of the cellular populations most involved in wound healing [32,33,36,37]. Silver, included in both formulations, showed a dose-dependent effect on the viability of both types of cells. Regardless of the cell type and time interval assessed, cell viability started to decrease when silver was used at concentrations at or above 7.5 mM. The collagen formulations proved to be more cytotoxic when compared with the same concentrations of silver-doped alginate formulations. Our results are in line with other findings, in which biomaterials containing proper concentrations of Ag were shown to be compatible with eukaryotic cells, such as fibroblasts [13,38].

## 5. Limitations

There are some limitations of our study that, if addressed, will provide more accurate data. First, the antibacterial activity was determined via agar diffusion method, which is one of the most accepted and common in vitro screening methods for testing the sensitivity of bacteria to antibiotics or other antimicrobial agents. However, the outcomes of this kind of assay are not always predictive for activity on live cells or animal tissues. Clinical limitations of in vitro testing of microorganism susceptibility have been known for years, as agar and broth systems cannot include all of the biologic variables and conditions found within the host environment [39,40]. For these reasons, further tests to confirm the antibacterial activity of these silver enriched scaffolds by in vitro and in vivo wound healing models are necessary. In addition, by using these models it would also be interesting to determine the duration of action of the matrices and their activity against microbial biofilms. However, despite its limitations, the agar diffusion method is still an invaluable tool for the screening of antimicrobial compounds.

Another area of improvement includes the quantification of silver release in culture media from the scaffolds using neutron activation analysis (NAA) or inductively coupled plasma mass spectroscopy (ICP-MS), which was not possible at this stage of the research. Nevertheless, we think that according to previous results on alginate, largely studied as drug delivery system and/or as cell carrier and recently also for gene immobilization, it is able to provide a sustained release of Ag, as it gradually degrades for several months [13]. This is also valid for collagen according to our previous works that proposed its use in different applications [24,41,42].

Furthermore, studies on the inflammatory activity of silver used to load scaffolds, including both in vitro macrophage activation tests and in vivo immunohistochemical analysis of biopsies of wound sites in animal skin defect model, should be done. We cannot even exclude that there may be silver anti-inflammatory activity, considering very recent papers that show that silver suppresses the production of pro-inflammatory cytokines and MMP, reducing inflammation [32,43].

## 6. Conclusions

This study showed, in vitro, the cellular response of fibroblasts and keratinocytes to silver-enriched alginate and collagen beads, and the ability of these scaffolds to inhibit bacterial growth, hypothesizing their use for favoring the healing of skin wounds.

The release of silver lactate and saccharinate from alginate and collagen scaffolds conferred antibacterial activity against bacterial strains usually found in infected wounds. From the results obtained, taking into consideration both the cell viability and the bacterial inhibition, we feel able to exclude formulations with concentrations of silver higher than 5 mM from possible use. Considering the higher aggressiveness of the collagen clusters on bacteria, compared to the alginate scaffold of equal concentration, we can hypothesize their use as the best antimicrobials. At the same time, the greater aggressiveness of the collagen scaffold on the vitality of fibroblasts should be taken into account. At all the concentrations tested, even the lowest, silver conveyed by collagen was effective as an antibacterial agent, so it might be used at a lower concentration to reduce both costs and cytotoxicity. Moreover, given the formulation of silver lactate was more effective than the formulation of silver saccharinate in terms of antibacterial activity, more economical and even less toxic on fibroblasts and keratinocytes, it seems the most promising for the purpose of application.

Skin lesions represent an ongoing challenge due to the high possibility of infection, which delays and compromises the healing process. The data obtained from these experiments are promising and could be the basis for future in vivo tests. In conclusion, the scaffolds developed are a promising approach for producing injectable or spreadable functional biomaterials for application in wound healing, thanks to their dual features combining biocompatibility and antibacterial properties.

## Figures and Tables

**Table 1:** Summary of the amount of silver contained in the beads.

			Alginate (7 µL)	Collagen (40 µL)
	mM	µg/mL	µg/mm^2^	µg/mm^2^
Ag lactate	150	3000	1.21	4.44
	15	300	0.12	0.44
	7.5	150	0.06	0.22
	3.75	75	0.03	0.11
Ag saccharinate	150	4500	1.81	6.66
	15	450	0.18	0.67
	7.5	225	0.09	0.33
	3.75	112.5	0.05	0.17

**Table 2:** IC_50_ calculated for silver enriched alginate and collagen beads against *S. epidermidis* and *P. aeruginosa* and expressed as mM.

	Alginate Beads	Collagen Beads
*S. epidermidis*/silver lactate	20.80	1.15
*S. epidermidis*/silver saccharinate	17.86	0.42
*P. aeruginosa*/silver lactate	23.42	1.01
*P. aeruginosa*/silver saccharinate	31.16	1.00
